# Hypnotic prescriptions in Japan may be shifting from benzodiazepine receptor agonists to other types of hypnotics, melatonin receptor agonists, and orexin receptor antagonists

**DOI:** 10.1002/pcn5.113

**Published:** 2023-06-19

**Authors:** Kensuke Usui, Daisuke Kikuchi, Noa Otsuka, Kouta Miyagi, Ryusuke Ouchi, Takashi Watanabe, Kouji Okada, Eiji Suzuki

**Affiliations:** ^1^ Division of Clinical Pharmaceutics and Pharmacy Practice Tohoku Medical and Pharmaceutical University Sendai Japan; ^2^ Department of Pharmacy Tohoku Medical and Pharmaceutical University Hospital Sendai Japan; ^3^ Faculty of Medicine Tohoku Medical and Pharmaceutical University Sendai Japan; ^4^ Division of Psychiatry Tohoku Medical and Pharmaceutical University Sendai Japan

Benzodiazepine hypnotics (BZDs) have been a popular treatment for insomnia since the 1960s, but they have various problematic side effects, including falls and delirium.^1^, ^2^ Non‐BZDs were developed right after BZDs and are BZD receptor agonists (BZD‐RAs) in action. In Japan, two other non‐BZD‐RA hypnotics were released, namely ramelteon (melatonin receptor agonist [MRA]) in 2010 and suvorexant (orexin receptor antagonist [ORA]) in 2014. Despite the existence of these non‐BZD‐RAs, according to an analysis using Medical Data Vision (MDV), 96.5% of the first‐prescribed sleep medications to Japanese insomnia patients in Japan (from 2012 to 2016) were BZD‐RAs.^3^ American studies reported similar trends during 1999–2010.^4^

In 2020, the US Food and Drug Administration (FDA) issued a warning about the risks of BZD‐RAs.^5^ Lemborexant, a novel ORA, was launched in 2020 in Japan. A relatively high proportion (66.7%) of patients across different groups can reportedly completely switch from BZD‐RAs to lemborexant.^6^, ^7^ This study investigated hypnotic prescription trend changes in Japan following the FDA warning and the launch of lemborexant, which can replace BZD‐RAs.

The study included inpatients and outpatients enrolled in the MDV analyzer, a hospital‐based claims database, from December 1, 2012 to November 30, 2021. The MDV analyzer contains anonymized claims data from 430 diagnosis–procedure combination hospitals and the total number of patients was 38.5 million (including deceased patients) as of October 31, 2021. This study included the following pharmaceuticals: BZDs estazolam, quazepam, triazolam, nitrazepam, haloxazolam, flunitrazepam, flurazepam, brotizolam, rilmazafone hydrochloride hydrate, and lormetazepam, non‐BZDs eszopiclone, zopiclone, and zolpidem tartrate, the only MRA was ramelteon, and the ORAs suvorexant and lemborexant. The annual change in rates of patients prescribed each hypnotic was analyzed using the Cochran Armitage trend test, and data from 2021 were analyzed using the *χ* ^2^ test. For convenience, data from December of the previous year to November of the current year were treated as data for the current year. Patients who were prescribed two or more hypnotics were counted for each drug.

From 2013 to 2021, hypnotic prescription rates changed nonsignificantly (2013 7.97%, 2021 7.98%; P = 0.9309), BZD prescriptions decreased significantly (2013 5.0%, 2021 2.9%; *P* < 0.0001), MRA (2013 0.3%, 2021 0.9%; *P* < 0.0001) and ORA (2013 0.0%, 2021 2.7%; *P* < 0.0001; Figure 1a) prescriptions increased significantly, and non‐BZD prescriptions changed nonsignificantly (2013 3.5%, 2021 3.3%; P = 0.5462). The absolute number of prescriptions for each hypnotic is shown in Table S1.

**Figure 1:**
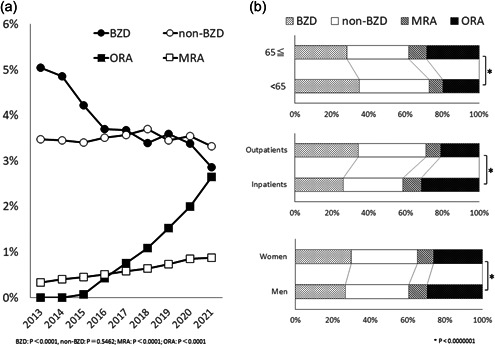
Trends in hypnotic prescriptions in Japan. (a) Annual changes in prescription rates for benzodiazepines (BZDs), non‐benzodiazepines (non‐BZDs), a melatonin receptor agonist (MRA), and orexin receptor antagonists (ORAs) in Japan. The total numbers of patients in 2013, 2014, 2015, 2016, 2017, 2018, 2019, 2020, and 2021 were 2,224,904, 2,215,412, 2,238,372, 2,223,210, 2,213,991, 2,216,877, 2,266,690, 2,072,925, and 2,087,112, respectively. (a) The rate of prescription for ORAs is gradually increasing and decreasing for BZDs. (b) Breakdown of hypnotic prescriptions in Japan in 2021 by BZDs, non‐BZDs, MRAs, and ORAs. From top to bottom, the results shown regards to comparisons of those aged 65 years and older versus those younger than 65 years, inpatients versus outpatients, and women versus men.

In 2021, hypnotic prescription rates were higher in patients aged 65 years and older (14.5%) than in those younger than 65 years (5.0%), in inpatients (33.5%) than in outpatients (4.5%), and in women (9.7%) than in men (9.4%) (all *P* < 0.0000001). When comparing prescription rates of different hypnotics in 2021 between those aged 65 years and older and those younger than 65 years, the former group showed lower rates for BZDs and non‐BZDs, and higher rates for the MRA and ORAs (all *P* < 0.0000001; Figure 1b). When comparing these rates between inpatients and outpatients, inpatients showed lower rates for BZDs and non‐BZDs and higher rates for the MRA and ORAs (all *P* < 0.0000001; Figure 1b). When comparing these rates between men and women, men showed lower rates for BZDs and non‐BZDs and higher rates for the MRA and ORAs (all *P* < 0.0000001; Figure 1b).

These results suggest that prescription rates for hypnotics are shifting from BZDs to MRAs and ORAs in Japan, although general hypnotic prescription rates remain similar. Hospitalized patients and older adults have a higher risk of experiencing some side effects (e.g., delirium and falls) of hypnotics,^8^, ^9^ and the high prescribing rates of the MRA and ORAs in our sample, especially for hospitalized patients and older adults, may indicate that the risks of hypnotics are becoming more widely recognized. Meanwhile, the issues that remain to be addressed in the near future are the prescription rates of non‐BZDs, which remained mostly unchanged, and of BZDs, which were prescribed more to women than to men, despite the intake of BZDs being known to be a risk in the perinatal period.^10^ It would be desirable for Japanese practitioners to actively switch to ORA prescriptions not only for first‐prescribed drugs but also for patients already prescribed BZDs.

Regarding study limitations, first, it was retrospective and observational, with reliance on a single database; the MDV was designed for patients treated at large acute‐care hospitals in Japan, so the results cannot be generalized to patients from other medical facilities. Second, the lack of data linkage between medical facilities in the MDV means that patients who were prescribed hypnotics at more than one facility may be included as separate patients.

## AUTHOR CONTRIBUTIONS

K.U. and E.S. designed the study, performed statistical analyses, analyzed and interpreted the data, and drafted and edited the manuscript. D.K., N.O., and K.M. collected the data. R.O., T.W., and K.O. performed the drafting and editing, and contributed to supervision. All authors read and approved the final manuscript.

## CONFLICT OF INTEREST STATEMENT

Eiji Suzuki received research grants from Eisai Co., Ltd. and Shionogi & Co., Ltd. Kensuke Usui, Daisuke Kikuchi, Noa Otsuka, Kouta Miyagi, Ryusuke Ouchi, Takashi Watanabe, and Kouji Okada have no conflicts of interest that are directly relevant to the content of this article.

## FUNDING INFORMATION

None

## ETHICS APPROVAL STATEMENT

This study conducted a retrospective analysis of data using a nationwide claims database managed by Medical Data Vision Co., Ltd., Tokyo, Japan. Because the data analyzed are anonymized and there was no direct patient involvement, ethical approval and patient consent was waived by the relevant institutional review boards. Medical Data Vision is a company that collects and uses patients' personal data, which are then anonymously processed in accordance with regulations laid down in the Japanese Act on the Protection of Personal Information and the European Union's General Data Protection Regulation.

## PATIENT CONSENT STATEMENT

N/A

## CLINICAL TRIAL REGISTRATION

N/A

## Supporting information

Supporting information.

## Data Availability

N/A
