# Supplementary material for: Hypnotic prescriptions in Japan may be shifting from benzodiazepine receptor agonists to other types of hypnotics, melatonin receptor agonists, and orexin receptor antagonists
Source: PCN Rep. 2023 Jun 19;2(2):e113. doi: 10.1002/pcn5.113 (PMC11114351; doi:10.1002/pcn5.113)
Supplement: Supplementary file 1 — Supporting information. [file PCN5-2-e113-s001.docx]

**Table S1. Number of patients prescribed hypnotics for each year from 2013 to 2021**

| **Year** | **2013** | **2014** | **2015** | **2016** | **2017** | **2018** | **2019** | **2020** | **2021** |
| --- | --- | --- | --- | --- | --- | --- | --- | --- | --- |
| **Total patients** | **2,224,904** | **2,215,412** | **2,238,372** | **2,223,210** | **2,213,991** | **2,216,877** | **2,266,690** | **2,072,925** | **2,087,112** |
| Benzodiazepines | 112,187 | 107,513 | 94,580 | 82,286 | 81,399 | 75,213 | 81,468 | 69,993 | 59,822 |
| Brotizolam | 60,182 | 59,602 | 55,475 | 47,368 | 46,587 | 43,098 | 47,212 | 40,205 | 33,921 |
| Rilmazafone hydrochloride hydrate | 15,548 | 14,114 | 9,664 | 7,490 | 7,498 | 6,024 | 7,135 | 5,531 | 4,077 |
| Triazolam | 12,711 | 11,769 | 9,822 | 9,125 | 9,057 | 8,569 | 9,378 | 8,167 | 7,357 |
| Flunitrazepam | 9,923 | 9,404 | 8,530 | 8,048 | 8,005 | 7,862 | 8,101 | 7,271 | 6,525 |
| Nitrazepam | 6,603 | 6,098 | 5,601 | 4,957 | 4,955 | 4,652 | 4,749 | 4,501 | 4,074 |
| Estazolam | 3,628 | 3,337 | 2,856 | 2,641 | 2,644 | 2,521 | 2,564 | 2,297 | 1,988 |
| Lormetazepam | 2,284 | 2,028 | 1,750 | 1,769 | 1,762 | 1,627 | 1,502 | 1,294 | 1,192 |
| Quazepam | 1,204 | 1,075 | 823 | 819 | 799 | 772 | 727 | 629 | 585 |
| Flurazepam | 95 | 79 | 52 | 64 | 80 | 80 | 76 | 81 | 85 |
| Haloxazolam | 9 | 7 | 7 | 5 | 12 | 8 | 24 | 17 | 18 |
| Non-benzodiazepines | 77,434 | 76,544 | 76,261 | 78,041 | 78,906 | 82,108 | 78,318 | 73,466 | 69,273 |
| Zolpidem tartrate | 56,483 | 54,960 | 54,573 | 54,707 | 54,605 | 54,509 | 51,810 | 45,965 | 41,538 |
| Zopiclone | 18,587 | 15,441 | 13,437 | 12,407 | 10,754 | 9,332 | 8,241 | 6,718 | 5,643 |
| Eszopiclone | 2,364 | 6,143 | 8,251 | 10,927 | 13,547 | 18,267 | 18,267 | 20,783 | 22,092 |
| Melatonin receptor agonist | 7,294 | 8,857 | 10,169 | 11,327 | 12,876 | 14,115 | 16,513 | 17,581 | 18,380 |
| Ramelteon | 7,294 | 8,857 | 10,169 | 11,327 | 12,876 | 14,115 | 16,513 | 17,581 | 18,380 |
| Orexin receptor antagonists | 0 | 0 | 1,707 | 9,415 | 16,718 | 24,126 | 34,492 | 41,469 | 55,339 |
| Suvorexant | 0 | 0 | 1,707 | 9,415 | 16,718 | 24,126 | 34,492 | 41,146 | 46,416 |
| Lemborexant | 0 | 0 | 0 | 0 | 0 | 0 | 0 | 323 | 8,923 |
